# *P*-value, compatibility, and S-value

**DOI:** 10.1016/j.gloepi.2022.100085

**Published:** 2022-09-12

**Authors:** Mohammad Ali Mansournia, Maryam Nazemipour, Mahyar Etminan

**Affiliations:** a Department of Epidemiology and Biostatistics, School of Public Health, Tehran University of Medical Sciences, Tehran, Iran; b Department of Ophthalmology, Medicine and Pharmacology, University of British Columbia, Vancouver, Canada

**Keywords:** *P*-value, Confidence interval, S-value, Compatibility interval, Significance

## Abstract

Misinterpretations of *P*-values and 95% confidence intervals are ubiquitous in medical research. Specifically, the terms significance or confidence, extensively used in medical papers, ignore biases and violations of statistical assumptions and hence should be called overconfidence terms. In this paper, we present the compatibility view of *P*-values and confidence intervals; the P-value is interpreted as an index of compatibility between data and the model, including the test hypothesis and background assumptions, whereas a confidence interval is interpreted as the range of parameter values that are compatible with the data under background assumptions. We also suggest the use of a surprisal measure, often referred to as the S-value, a novel metric that transforms the *P*-value, for gauging compatibility in terms of an intuitive experiment of coin tossing.

## 1 Introduction

A recent multicenter randomized trial at 130 sites in 18 countries hypothesized that ticagrelor, in combination with aspirin for 1 month, followed by ticagrelor alone, improves outcomes after percutaneous coronary intervention compared with standard antiplatelet regimens [1]. The primary endpoint at 2 years was a composite of all-cause mortality or new Q-wave myocardial infarction. The intention-to-treat rate ratio (RR) estimate using the Mantel-Cox method was 0.87 [95% confidence interval (CI): 0.75–1.01] with two-sided *P*-value of 0·073. The authors concluded that “In our multicenter randomized trial, ticagrelor in combination with aspirin for 1 month followed by ticagrelor alone for 23 months was not superior to standard 1-year dual antiplatelet therapy followed by aspirin monotherapy in terms of the composite endpoint of all-cause mortality or new Q-wave myocardial infarction after percutaneous coronary intervention” [1]. This conclusion is based on comparing the *P*-value of 0.073 to the cutoff default value of 0.05. Also, the paper freely uses the term “significantly” including the expression of “did not differ significantly between … groups” four times.

Such misinterpretations of *P*-value based on the cutoff value of 0.05 and ignorance of the association measure estimate and 95% confidence interval are not uncommon in medical research, which are a consequence of using overconfidence terms such as significance or confidence. In this paper, we argue that *P*-values and confidence intervals should be interpreted as compatibility measures of different values of parameters with data, and suggest using an alternative measure known as the S-value, which better facilitates the compatibility view.

## 2 *P*-value as a measure of compatibility

The *P-value* is often defined as the probability of the observed or more extreme results if the *test hypothesis* is true. This definition implicitly assumes some *background assumptions* including population distribution of the outcome variable (e.g., Normal distribution), random sampling or randomization of the participants, random measurement error in the exposure and outcome variables, and no bias in the design, execution, analysis, and reporting. In fact, a statistical-testing procedure tests both the test hypothesis and background assumptions, which we refer to as the *model*. The *P*-value is an index of *compatibility* between the data and the model, which varies between 0 (completely incompatible) to 1 (completely compatible) [[2], [3], [4], [5], [6]]. For a sufficiently small *P*-value, we conclude that the model is incorrect, that is, either the test hypothesis or background assumptions or both are incorrect; otherwise we can assume that a rare event has occurred [2]. Thus a very small *P*-value doesn't necessarily indicate a false test hypothesis if some background assumptions are violated. However, for a sufficiently large *P*-value, we can only say that the data are *compatible* with the model predictions. However, we cannot conclude that the model is correct as the *P*-value is not an index of *support* for the tested model [2,3,7]. In clinical studies, there is no guarantee that the background assumptions embedded in the model are correct, and in fact many assumptions are often violated in practice. In the example mentioned above, the model assumes absence of all Cochrane biases [8] including selection bias, performance bias, detection bias, attrition bias, and reporting bias as well as random confounding [9][[19], [20], [21], [22]]. Also the Mantel-Cox test used in the paper is based on the following assumptions: [10][23] censoring is independent of the outcome, the survival probabilities do not vary with follow-up time, and the events occurred at specified times. Censoring due to deaths (about 3% in each group) and lack of blinding may violate some of these assumptions. Moreover, adherence to the allocated intervention was not perfect and some participants in both groups did not receive or complete the allocated intervention, so the analysis was intention-to-treat (ITT). The ITT approach does not invalidate the hypothesis testing, however [8].

## 3 S-value

To avoid misinterpretations of the *P*-value, we suggest transforming it to a quantity known as the *Shannon-information* or *surprisal* or *self-information* called *S-value* [[3], [4], [5], [6],[11], [12], [13]] (see Appendix 1):

$$S-\text{value}=-\log_{2}\left(P-\text{value}\right)=-\frac{\log_{e}\left(P-\text{value}\right)}{\log_{e}2}$$

**Fig. 1:**
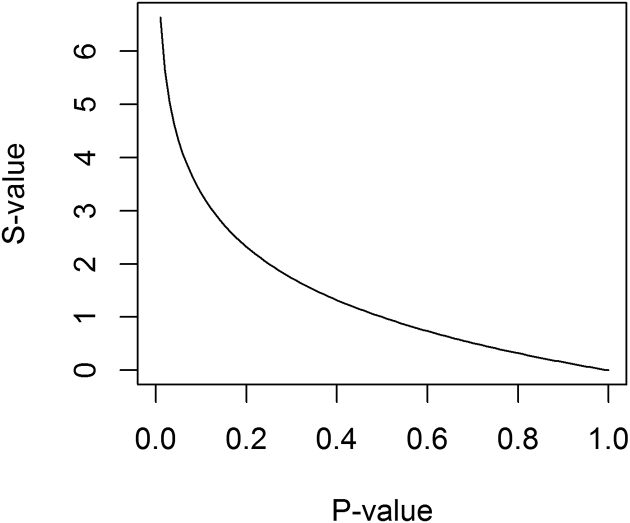
S-value vs. P-value.

With base 2 for the logarithm, the S-value is scaled in *bits* (binary digits) of information, where “bit” refers to the information capacity of a binary (0,1) digit. Thus the S-value is the number of bits of information in the data against the model, including background assumptions and the test hypothesis. Fig. 1 shows that the S-value exponentially increases as the *P*-value goes to zero. In the limits, the S-value = 0 when the P-value = 1, which implies that the data provide no information against the model, but as for *P*-value = 1, we cannot conclude that the model is correct the S-value approaches infinity when the *P*-value approaches to zero, which indicates that the data provide infinite information against the model, leading one to a more decisive conclusion that the model is incorrect.

Unlike the P-value, the S-value has an intuitive interpretation in a physical experimental coin tossing. Suppose we are concerned about fairness of a coin, so we toss it 4 times and the result turns out to be 4 heads. The *P*-value would be ${\left(\frac{1}{2}\right)}^{4}$, and the S-value 4, which conveys the same evidence against the model as seeing all heads in 4 independent tosses of a coin against the hypothesis that the coin is fair [3]. As an example, the S-value of 4.3 bits corresponding to an observation of *P*-value = 0.05 is hardly more surprising than seeing all heads in 4 fair tosses. This shows that the common dichotomization of *P*-value at 0.05 is an overstatement of evidence against the model as the amount of information that a P-value = 0.05 conveys is small [3,4]. Significance testing has been popular simply due to its simplicity as it has allowed researchers and clinicians to make decisions based on the cutpoint of 0.05.

In fact, more stringent cutpoints are used outside the health sciences. For example, the 5-sigma criterion for discovery in physics as used for Higgs boson particle corresponds to a one-sided *P*-value of about 1 per 3.5 million with a corresponding S-value of 21.7 bits [14]. Another advantage of the S-value is that log scaling makes information additive, e.g., two independent studies with the same test hypothesis yielding a *P*-value of 0.05 provides an S-value of 4.3 + 4.3 = 8.6 bits of information against the model. Finally, the S-value resolves some misconceptions about the *P*-value, as shown in Table 1 [3,[15], [16], [17]]. The reported P-value of 0.073 in the case study translates to an S-value of 3.8 bits, which is hardly less surprising than seeing all heads in 4 fair tosses. This S-value clearly suggests that it is unjustified to differentially treat *P*-values of 0.073 and 0.05, as the S-value, unlike the P-value, is a metric that does not contain any cutpoint.

**Table 1:** Some misinterpretations of *P*-values and their resolution using S-values.

Misinterpretations of P-values	Clarification by S-values
P-value is the probability that the result is due to chance	S-value is not bounded to be between 0 and 1 so it is not confused with this probability
P-value is an error probability resembling the alpha level	S-value is not bounded to be between 0 and 1 so it is not confused with this probability
Large P-values indicate test hypothesis is plausible and small *P*-values indicate test hypothesis is implausible	S-values provide refutational information against the model including both background assumptions and test hypothesis
A P-value <0.05 implies test hypothesis is false and a P-value >0.05 implies test hypothesis is correct	S-value has an intuitive interpretation based on observing all heads in fair coin tossing to gauge the evidence against the model without any reference to an arbitrary cutpoint S-value shows that the amount of information in the *P* = 0.05 is small (only 4.3 bits)
Equal intervals in P-value represent equal changes in the evidence as measured by the SD change	Equal intervals in S-value represent equal changes in the evidence as measured by the information

## 4 Testing alternative hypotheses

Researchers tend to report P-values only for the null hypothesis, which often corresponds to no association between two variables in the population. However, they can and should test alternative hypotheses, especially those that correspond to minimal clinically important differences [18], and compare the compatibility of different parameter values with the data [3]. As an example, the *P*-value for the RR of 0.8 for the primary endpoint in our example is 0.27 (please see Appendix 2 for the computations) which translate to an S-value of −log_2_0.27 = 1.9 bits. Therefore, a 20% reduction in the rate of the primary endpoint of the study is more compatible with the data than the rate ratio of 1 (S-value = 3.8). Also, the paper reports RR of 0.8 [95% CI: 0.60–1.07] with a *P*-value of 0.14 for the endpoint of new Q-wave myocardial infarction with a corresponding S-value equaling −log_2_0.14 = 2.8 bits. The authors concluded that “The frequency of … new Q-wave myocardial infarction … did not differ significantly between groups”. However, we can verify that the *P*-value for the RR of 0.75 equals 0.66 with an S-value of−log_2_0.66 = 0.60 bits. Thus, the information against RR of 1 is 2.2 bits higher than that for RR of 0.75, which spoils the conclusion of the paper.

## 5 Compatibility intervals

The 95% confidence interval is often interpreted as the range of values which include the parameter of interest with the probability of 95%. However, in the presence of biases, the background assumptions are not met (e.g., the assumptions of random sampling and randomization are violated in observational studies) and thus confidence intervals should be more accurately termed as *overconfidence intervals.* We prefer to use the term *compatibility intervals* with the following interpretation: The 95% confidence interval includes the range of values which are compatible with the data, that is, statistical testing of values provides no >4.3 bits of information against them assuming the background assumptions are correct. In our case-study, statistical testing provides no >4.3 bits of information against the rate ratios in the range of 0.75–1.01 (4.3 bits information are against the rate ratio limits of 0.75 and 1.01). Moreover, there is no information against 13% decrease in the rate of the primary endpoint among the experimental group compared to the control group (RR = 0.87, *P*-value = 1, and S-value = 0).

## 6 Conclusion

The P-value should be interpreted as an index of compatibility between the data and the model, including the test hypothesis and background assumptions. The confidence interval should be named compatibility interval, and interpreted as the range of values which are compatible with the data. The S-value represents the information of the data against the model, facilitating the compatibility interpretation. Moreover, it is not subject to many misinterpretation of the *P*-value, and should be used in practice along with the P-value and compatibility interval. This is especially the case when interpreting results of clinical studies.

## Appendix 1 S-value

The *S-value*, the *Shannon-information*, *surprisal*, or *self-information* is a logarithmic transformation of P-value: $S-\text{value}=-\log_{2}\left(P-\text{value}\right)=-\frac{\log_{e}\left(P-\text{value}\right)}{\log_{e}2}$. As S-value is calculated using base-2 logarithm, its units are called bits (binary digits) of information where “bit” refers to the information capacity of a binary (0, 1) digit. The first integer larger than S-value is the number of binary digits needed to encode $\frac{1}{P-\mathrm{value}}$ e.g., the S-value for *P*-value = 0.05 is 4.3 and $\frac{1}{0.05}=20$ is written in binary code as 10,100 with 5 digits, because 16 + 0 + 4 + 0 + 0 = 20.

Unlike P-value, the S-value has an intuitive interpretation: it conveys the same information or evidence against the entire model as seeing all heads in k independent tosses of a coin conveys against the hypothesis that the coin is fair where k is the nearest integer to the S-value. As an example, the S-value of 4.3 bits corresponding to an observation of *P*-value = 0.05 is hardly more surprising than seeing all heads in 4 fair tosses with the probability of ${\left(\frac{1}{2}\right)}^{4}=\frac{1}{16}$. We note that, the *expected information*, called *Shannon entropy*, which is the average of S-values against the entire model is 1.44 bits, so by chance alone we should expect to see 1 or 2 bits of information.

The 95% confidence interval, which we call compatibility interval, can be interpreted using S-value. The 95% compatibility interval includes the range of values for which statistical testing supplies no >4.3 bits of information against assuming the background assumptions are correct. Also the study power can be defined using the S-value concept: With an alpha level of 0.05, the power is the probability of obtaining at least 4.3 bits of information against the model including the test hypothesis and background assumptions if the alternative hypothesis (often corresponding to a minimal clinically important difference) is correct.

Information penalization should be performed for data-driven selection and multiple comparisons. As an example, two-sided *P*-value, the double of the smaller one-sided P-value, is the default for statistical testing in medical research as the direction of the violation of test hypothesis is often not known. Doubling subtracts 1 bit of information from the S-value: the information of 1 is a penalty for the data pick the test direction. As an example, Z = 1.79 in our case-study yields two one-sided *P*-values: 0.0367, and 0.9633. The S-value for the smaller P-value, 0.0367, is 4.8, but we cannot exclude the possibility that the experimental treatment is worse than the control treatment. So we have to double the smaller P-value to obtain *P* = 0.073 which is translated to S = 3.8: we used up 1 bit of information to let the data choose the test direction. As another example, the Bonferroni adjustment preserves the alpha level, the probability of making at least one type-1 error, for multiple testing by multiplying *P*-values by K, the number of comparisons. The information penalty is then log_2_*K* (e.g., 2 if K = 4).

## Appendix 2 Testing alternative hypotheses

The Wald chi-square test statistic can be calculated for testing alternative hypothesis H_A_: θ = θ_1_, using the point estimate T with estimated standard error S as follows:

$$P-\mathrm{value}=P\left(\chi^{2}\left(1\right)>{\left(\frac{T-\theta_{1}}{S}\right)}^{2}\right)$$

where *χ*^2^(1) is a chi-squared random variable with degree freedom of 1. For example, in the case study, θ_1_ = ln(0.8),T = ln(0.87), and S = $\frac{\ln\left(1.01\right)-\ln\left(0.75\right)}{2\times 1.96}=0.076$, and so

$$P-\mathrm{value}=P\left(\chi^{2}\left(1\right)>{\left(\frac{\ln\left(0.87\right)-\ln\left(0.8\right)}{0.076}\right)}^{2}\right)=P\left(\chi^{2}\left(1\right)>1.22\right)=0.27$$

## References

[1] Vranckx P., Valgimigli M., Jüni P., Hamm C., Steg P.G., Heg D. (2018). Ticagrelor plus aspirin for 1 month, followed by ticagrelor monotherapy for 23 months vs aspirin plus clopidogrel or ticagrelor for 12 months, followed by aspirin monotherapy for 12 months after implantation of a drug-eluting stent: a multicentre, open-label, randomised superiority trial. Lancet.

[2] Greenland S., Senn S.J., Rothman K.J., Carlin J.B., Poole C., Goodman S.N. (2016). Statistical tests, P values, confidence intervals, and power: a guide to misinterpretations. Eur J Epidemiol.

[3] Greenland S. (2019). Valid P-values behave exactly as they should: some misleading criticisms of P-values and their resolution with S-values. Am Stat.

[4] Greenland S. (2017). Invited commentary: the need for cognitive science in methodology. Am J Epidemiol.

[5] Rafi Z., Greenland S. (2020). Semantic and cognitive tools to aid statistical science: replace confidence and significance by compatibility and surprise. BMC Med Res Methodol.

[6] Greenland S., Mansournia M.A., Joffe M. (2022). To curb research misreporting, replace significance and confidence by compatibility: a preventive medicine golden jubilee article. Prev Med.

[7] Amrhein V., Greenland S., McShane B. (2019).

[8] Mansournia M.A., Higgins J.P.T., Sterne J.A.C., Hernán M.A. (2017). Biases in randomized trials: a conversation between Trialists and epidemiologists. Epidemiology.

[9] Greenland S., Mansournia M.A. (2015). Limitations of individual causal models, causal graphs, and ignorability assumptions, as illustrated by random confounding and design unfaithfulness. Eur J Epidemiol.

[10] Bland J.M., Altman D.G. (2004). The logrank test. BMJ..

[11] Shannon C.E. (1948). A mathematical theory of communication. Bell Syst Tech J.

[12] Good I.J. (1956). The surprise index for the multivariate normal distribution. Ann Math Stat.

[13] Cole S.R., Edwards J.K., Greenland S. (2021). Surprise!. Am J Epidemiol.

[14] Horton R. (2015). Offline: what is medicine’s 5 sigma. Lancet..

[15] Mansournia M.A., Collins G.S., Nielsen R.O., Nazemipour M., Jewell N.P., Altman D.G. (2021). CHecklist for statistical assessment of medical papers: the CHAMP statement. Br J Sports Med.

[16] Mansournia M.A., Collins G.S., Nielsen R.O., Nazemipour M., Jewell N.P., Altman D.G. (2021). A CHecklist for statistical assessment of medical papers (the CHAMP statement): explanation and elaboration. Br J Sports Med.

[17] Altman D.G., Bland J.M. (1996). Absence of evidence is not evidence of absence. Aust Vet J.

[18] Nielsen R.O., Bertelsen M.L., Verhagen E., Mansournia M.A., Hulme A., Møller M. (2017). When is a study result important for athletes, clinicians and team coaches/staff?. Br J Sports Med.

[19] Mansournia MA, Nazemipour M, Etminan M (2022). Interaction contrasts and collider bias. American Journal of Epidemiology.

[20] Etminan M, Collins GS, Mansournia MA (2020). Using causal diagrams to improve the design and interpretation of medical research. Chest.

[21] Mansournia MA, Nazemipour M, Etminan M (2021). Causal diagrams for immortal time bias. International journal of epidemiology.

[22] Etminan M, Brophy JM, Collins G, Nazemipour M, Mansournia MA (2021). To adjust or not to adjust: the role of different covariates in cardiovascular observational studies. American Heart Journal.

[23] Mansournia MA, Nazemipour M, Etminan M (2022). A practical guide to handling competing events in etiologic time-to-event studies. Global Epidemiology.

